# Aspirin in the 21st century—common mechanisms of disease and their modulation by aspirin: a report from the 2015 scientific conference of the international aspirin foundation, 28 August, London, UK

**DOI:** 10.3332/ecancer.2015.581

**Published:** 2015-10-13

**Authors:** Tom Smith, Pippa Hutchison, Karsten Schrör, Joan Clària, Angel Lanas, Paola Patrignani, Andrew T Chan, Farhat Din, Ruth Langley, Peter Elwood, Andrew Freedman, Ron Eccles

**Affiliations:** 1 The Croft, Pinwherry, Girvan, Ayrshire, Scotland KA26 0RU, UK; 2 International Aspirin Foundation, 34 Bower Mount Road, Maidstone, Kent ME16 8AU, UK; 3 Institut für Pharmakologie und Klinische Pharmakologie, Heinrich-Heine-Universität, Düsseldorf, D-40225 Düsseldorf, Germany; 4 Hospital Clínic-University of Barcelona, Barcelona, Catalonia 08036, Spain; 5 Service of Digestive Diseases, University Hospital, University of Zaragoza, IIS Aragon, CIBERehd, Zaragoza, Spain; 6 Department of Neuroscience, Imaging and Clinical Sciences, “G. d’Annunzio” University, 66100 Chieti, Italy; 7 Clinical and Translational Epidemiology Unit and Division of Gastroenterology, Massachusetts General Hospital, Boston, MA, USA; 8 Institute of Genetics and Molecular Medicine, University of Edinburgh, UK; 9 MRC Clinical Trials Unit, University College London, Institute of Clinical Trials & Methodology, London, UK; 10 Cochrane Institute, Dept. of Primary Care and Public Health, Cardiff University, UK; 11 Cardiff University School of Medicine, Heath Park, Cardiff CF14 4XN, UK; 12 Common Cold Centre, Cardiff University UK CF10 3AX, UK

**Keywords:** aspirin, cancer, Lynch syndrome, Hughes syndrome, diabetes, HIV

## Abstract

Professor Peter Rothwell of Oxford University chaired the annual Scientific Conference of the International Aspirin Foundation in London on 28 August 2015. It took the form of four sessions.

Aspirin has more than one action in its effects on disease. Its acetylation of cyclooxygenase 2 (COX-2) in platelets leads to the blockade of pro-inflammatory chemicals and generation of anti-inflammatory mediators and increase in nitrous oxide (NO) production, which helps to preserve arterial endothelium. But platelets are not its only target. There is now evidence that aspirin has a direct antitumour effect on intestinal mucosal cells that block their potential transformation into cancer cells.

Randomised placebo-controlled trials (RCTs) in people with histories of colorectal neoplasia have shown that aspirin reduces the risk of recurrent adenomas and reduces long-term cancer incidence in patients with Lynch syndrome. Among women given aspirin for cardiovascular disease, there were fewer cancers than in those given placebo. Epidemiological evidence has suggested that aspirin treatment after cancer is diagnosed reduces the incidence of metastases and prolongs survival, and long-term studies of anticancer treatment with aspirin are under way to confirm this.

Apart from cancer studies, aspirin use is now firmly established as treatment for antiphospholipid syndrome (Hughes syndrome) and is being used to prevent and treat the heightened risk of cardiovascular disease in diabetes mellitus and in patients with HIV.

It remains, of course, a first line treatment of choice in acute pain: randomised trials of its use for wisdom tooth extraction pain have shown that it is more effective than placebo and more useful than paracetamol.

In the opening session of the conference, on the mechanisms of action of aspirin, the first speaker, Dr Karsten Schrör of the Heinrich-Heine-University of Düsseldorf, explained that aspirin contains two pharmacologically relevant moieties: the reactive acetyl group and salicylate. At doses of 75- 325 mg, most, perhaps even all, of its clinical actions such as prevention of thrombosis and of colorectal cancer are due to target structure acetylation [1]. The best known pharmacological action, which occurs with the low daily dose of 75 mg, is on cyclo-oxygenase-1 (COX-1) in platelets with subsequent inhibition of platelet-dependent thromboxane formation. This is the key to blockade of a cascade of platelet functions. In addition to inhibition of aggregation this includes inhibition of the release of pro-inflammatory and mitogenic chemicals such as P-selectin, sphingosine-1-phosphate (SIP), vascular endothelial growth factor (VEGF) and others. Aspirin acetylating of COX-2 allows generation of anti-inflammatory and inflammation resolving lipid mediators, such as the “aspirin-triggered lipoxins” (ATL). A third aspirin action is acetylation of eNOS, which increases nitric oxide (NO) production. This directly helps to preserve arterial endothelium by improving oxygen defense [2]. The more detailed investigation of these and possible identification of new acetylation targets is currently of considerable interest to pharmacologists to explain the anticancer effect of the compound.

Continuing with the mechanism of action theme, Professor Joan Claria, of the University of Barcelona, stated that it may involve the generation of a series of eicosanoids called ASA-triggered lipoxins, produced when aspirin acetylates COX-2 [3]. Other possibilities are the newly discovered ASA-triggered resolvins derived from omega-3 fatty acids. They share anti-inflammatory and ‘pro-resolution’ properties, which are likely to be part of aspirin’s benefits [4].

Aspirin increases the risk of bleeding and the reason was addressed by Professor Angel Lanas of the University of Zaragoza. Bleeding in the upper, and to a lesser extent, the lower gut, is related to interference with the coagulation cascade instituted by platelets. However, there are other factors that make people more likely to bleed with aspirin. Important among them are a history of peptic ulcer, older age, combining aspirin with NSAIDs, and higher aspirin dose. Professor Lanas described prevention strategies to lower the risk of bleeding, along with the use of proton pump inhibitors and perhaps *H Pylori* eradication [5]. The way forward may be with the former, as the benefit of the latter is still to be proven.

Aspirin’s benefits in colorectal cancer may not be solely due to its actions on platelets. Professor Paola Patrignani, of “G d’Annunzio” University, Chieti, who participated by Skype from Italy, proposed that aspirin has benefits other than those on platelets that add to its effects on carcinogenesis in the bowel [5]. She admitted that the platelet effect is crucial. Tumours metastasize because cancer cells are transported by the circulation to distant sites by their adherence to platelets. By preventing platelet-cancer cell interactions this mechanism is blocked, and circulating leucocytes ‘recognize’ the neoplastic cells and destroy them [6].

However, aspirin, even at low-doses, almost certainly also acts directly on the colorectal mucosal cells. Professor Patrignani and her colleagues have developed a new assay which allows the detection of the extent of acetylation of COX-1 in platelets and colorectal mucosa by the administration of low-dose aspirin [7]. They showed that aspirin acetylates COX-1 in platelets but also in rectal mucosa and this effect is associated with inhibition of prostaglandin E2 and changes of the rectal mucosal phenotype in a way that would delay or prevent the early development of colorectal cancer.

Session two concentrated on the clinical evidence of the effect of aspirin on cancer. The first speaker, Professor Andrew Chan, of Massachusetts General Hospital, said that the evidence that it is linked to a lower risk of colorectal cancer is remarkably consistent. Five placebo-controlled randomised trials (RCTs) in people with histories of colorectal neoplasia showed that it reduced the risk of recurrent adenomas, which are the precursors of most cancers. Other RCT confirmation of aspirin’s protection comes from long-term follow-up of people with the Lynch hereditary colorectal cancer syndrome. Furthermore, in women randomised to aspirin or placebo for the primary prevention of cardiovascular disease, there were fewer colorectal cancers among those given aspirin than among those taking placebo.

The most recent results in a secondary cardiovascular event prevention trial even suggest that the cancer prevention extends to cancers beyond the colorectal area. Professor Chan concluded that there may well be a role for aspirin in the prevention of other cancers.

Dr Farhat Din, of Edinburgh University, acknowledged the epidemiological and RCT evidence that aspirin has striking chemoprotective properties against colorectal cancer. Not only does it reduce incidence and mortality, but it also improves survival in patients who already have the disease. She sets herself the task of understanding why it does so. Environmental factors account for more than half the variation in colorectal cancer risk, one of which is obesity. The risk of developing colorectal cancer is 30% lower with regular physical activity and around 10% higher in the obese overall. This obesity-related risk is even higher in men in whom it is raised by from 30% to 70%. Therefore, Dr Din’s team, in keeping with other researchers, suggests that imbalance of energy and metabolism pathways may initiate and then promote colorectal cancer.

Dr Din’s team therefore studied the mTOR pathway that is pivotal in controlling cell survival, the regulation of metabolism and energy homoeostasis. mTOR integrates stimuli in the cell from growth factors, nutrient and signalling pathways. In colorectal cancer, energy imbalance is due to environmental and genetic risks converge on abnormal signalling pathways. These are likely to alter colorectal crypt metabolism and growth towards adenoma and then to carcinoma. Aspirin targets several of these pathways.

Dr Din’s group attribute the antitumour activity of aspirin to its potent inhibition of mTOR signalling and of its activation of AMP kinase phosphorylation, which also inhibits mTOR [17]. An early stage of induction of cancer is the initiation of protein translation. Dr Din showed that aspirin is key to blocking translation initiation and elongation, a novel insight into the effects of aspirin in colorectal cancer.

The second session concluded with a combined presentation by Dr Ruth Langley of London and Professor Peter Elwood of Cardiff on the opportunities for using aspirin to treat cancer. They reviewed the epidemiological evidence showing the benefits of aspirin after cancer is diagnosed in preventing metastases and prolonging survival. They discussed the evidence for there being specific tumour mutations that would be biomarkers of response to aspirin. They also discussed the frequency of gastrointestinal bleeding and the absence of evidence that fatal gastrointestinal bleeding is increased by low-dose aspirin—an important factor of relevance to the reluctance of doctors to prescribe low-dose aspirin. Finally, they presented planned and ongoing clinical trials aiming to establish a role (or not) for aspirin in the treatment of colorectal cancer.

The third session moved away from cancer to other diseases in which aspirin has profound effects. More than 30 years ago, Professor Graham Hughes of London described antiphospholipid syndrome (APLS) for the first time and it has been named after him as Hughes syndrome. Unfortunately, Professor Hughes was unable to attend, but in his notes he explained that it is an autoimmune disease that causes venous and arterial thrombosis. Commonly, it causes migraine, memory loss, epilepsy, angina and recurrent miscarriage. It is the commonest treatable cause of pregnancy loss.

The initial studies of aspirin in APLS-affected women increased the previous successful pregnancy rate from under 20% to over 90%. It is now combined with low-molecular-weight heparin for APLS-positive women with severe miscarriage histories and/or previous thrombosis [9]. Professor Hughes’ notes ended with a plea for more widespread testing for APLS to help other vulnerable subsets of patients.

Professor Carlo Patrono, of the Catholic University School of Medicine, Rome, took over the theme of aspirin in diseases other than cancer with a report on his research into its use in diabetes mellitus. He explained that in both types 1 and 2 diabetes, there is particularly high thromboxane synthesis, so that platelets are primed to over-react to coronary plaque fissuring or rupture, greatly increasing thrombosis risk [10]. There is, however, evidence that in some patients with type 2 diabetes, the level of thromboxane may recover rapidly after a dose of aspirin and a once daily regime may be suboptimal for such patients. Professor Patrono asked for RCTs to test a personalised antiplatelet regimen (such as twice daily doses). Even with dual or triple antiplatelet regimens given for acute coronary syndromes the residual risk of further events is substantial [11]. We need further investigation to find ways of reducing such risks.

Dr Andrew Freedman, of Cardiff Medical School, highlighted an aspect of HIV that is perhaps underestimated by clinicians. Before anti-HIV combination therapy (HAART) started in the 1990s patients with it progressed to terminal disease in around 10 years, and it was always fatal. However, now, if it is not diagnosed too late, most patients with HIV can expect to live into old age. Nevertheless, they still have a higher than normal death rate from other diseases, such as malignancies, cardiovascular disease, renal, hepatic or neurological diseases [12]. They are becoming commoner in HIV patients as with their longer survival and older age.

The evidence suggests that people on HAART are in a state of heightened immune activation and inflammation, both of which probably contribute to their higher morbidity and mortality [13]. As aspirin may well be beneficial in such circumstances, Dr Freedman instituted a short pilot study in which aspirin was given for one week to patients with HIV and to controls. The patients with HIV had at baseline significantly higher platelet activation than the controls [14]. With aspirin platelet aggregation and markers of cellular activation, both diminished. The latter included sCD14, lowering of which protects the colorectal mucosal barrier against the action of gram-negative bacteria.

Serum levels of sCD14 and other inflammatory markers are independent predictors of mortality from non-AIDS events such as myocardial infarction, stroke, malignancies and serious bacterial infections, and of overall mortality. Dr Freedman described three trials, one complete and two ongoing, which should soon indicate the value of aspirin in surviving patients with HIV.

The session ended with the use of aspirin in its original indication—acute pain. Professor Ron Eccles of the Common Cold Centre, Cardiff explained that as aspirin has been freely available for more than 100 years as an analgesic with unquestioned efficacy and lack of addictive properties, there has been little demand to show its effects in clinical trials. However, RCTs of aspirin in acute pain, in headache and migraine, common cold and ‘flu, muscle aches and pains, menstrual pain, toothache and post-tooth extraction pain, and the pains of arthritis together provide hard clinical evidence for its efficacy [15].

One pain model that has been standardised for clinical trials is that following wisdom tooth extraction: Its timing and intensity is predictable and it is a common dental procedure. These studies show that aspirin is more effective than placebo and more useful than paracetamol [16].

Professor Eccles added that the formulation of aspirin products markedly changes its absorption and pharmacokinetics. Simple aspirin is absorbed more slowly than effervescent and buffered aspirin. Times to peak concentration vary from 33 to 83 min with the standard tablet to only 18 min in the case of micronised tablets with an effervescent nucleus, giving the prescriber an extra choice in the case of acute and severe pain.

## Conclusion

Although aspirin has been in clinical use for more than a century, there is still much to be learned about its future uses, particularly in cancer and in patients who are at particularly high risk of cardio- and cerebrovascular diseases—such as those with diabetes mellitus and long-term survivors of HIV infection. This conference has mapped out its future uses. We await the results of the current clinical trials with some hope and optimism.

## Conflicts of interest

PE occasionally presents at meetings and conferences organised by Bayer HealthCare, and has received an occasional small honorarium. Dr Tom Smith is a freelance medical journalist, a retired general practitioner and pharmaceutical physician. He was paid a fee by The Aspirin Foundation for reporting on this meeting, but the Foundation had no editorial say in the text.
